# Hyaluronic acid increases MMP-2 and MMP-9 expressions in cultured trabecular meshwork cells from patients with primary open-angle glaucoma

**Published:** 2012-05-04

**Authors:** Mao-Sheng Guo, Yu-Yu Wu, Zong-Bao Liang

**Affiliations:** The Second Affiliated Hospital, Fujian Medical University, Quanzhou, China

## Abstract

**Purpose:**

A depletion of hyaluronic acid (HA) in patients’ eyes may be associated with primary open-angle glaucoma (POAG), but the exact mechanism remains unclear. We investigated the effect of HA on the expression of matrix metalloproteinases (MMP-2 and MMP-9) in cultured trabecular meshwork cells.

**Methods:**

Trabecular meshwork cells were cultured from trabecular tissues obtained from the POAG patients aged 23 to 45. The mRNA expression of *MMP-2* and *MMP-9* was determined by reverse transcription-polymerase chain reaction, and the protein expression of MMP-2 and MMP-9 by gelatin zymography analysis and qualified by the gel electrophoresis image analysis in different HA concentrations.

**Results:**

The expression of MMP-2 and MMP-9 by the two methods significantly increased with HA concentration in a dose–response manner. Mean values of the MMP-2 expression by the gelatin zymography analysis were 176, 264, 353, and 448 mg/ml, and mean values of the MMP-9 expression were 547, 659, 895, and 1,147 mg/ml, for HA concentration level of 0, 1, 3, and 6 mg/ml, respectively.

**Conclusions:**

In POAG trabecular meshwork cells, the level of HA concentration increases the activities of MMP-2 and MMP-9. The lack of HA in aqueous humor can result in a reduction in activities of MMPs and therefore may be involved in the pathogenesis of POAG.

## Introduction

Glaucoma is one of the most common causes of blindness and it often leads to irreversible damage of the optic nerve [1]. Although the pathogenesis of primary open-angle glaucoma (POAG) is not fully understood, elevated intraocular pressure remains the leading risk factor. Numerous studies suggest that the elevated intraocular pressure may be attributable to the change and structure of trabecular meshwork extracellular matrix (ECM) that limits the aqueous outflow [2-4]. Matrix metalloproteinases (MMPs) are zinc-dependent endopeptidases. A reduction of MMPs in aqueous humor can alter the balance between MMPs and the tissue inhibitors of metalloproteinases (TIMPs). This imbalance can lead to an accumulation of ECM and subsequently the development of POAG [5].

Hyaluronic acid (HA) is one of the major components of the ECM and may attribute to the filtration function of the trabecular meshwork. A study by Knepper et al. [6] found that the amount of hyaluronic acid in the POAG trabecular meshwork was 77% less than that in the normal trabecular meshwork (p*<*0.02), and HA was detected in only 4 of the 10 studied POAG trabecular meshworks. The authors concluded the depletion of HA may increase aqueous outflow resistance in the trabecular meshwork of POAG patients.

In light of these findings, we hypothesized that in the trabecular meshwork cells of the POAG patients, reduced HA can lead to down-regulation of MMPs and therefore contribute to the disruption of ECM and subsequently the development of POAG. We investigated the effects of different level of HA concentrations on mRNA and protein expressions of MMP-2 and MMP-9 in cultured trabecular meshwork cells using reverse transcription-polymerase chain reaction (RT–PCR) method and gelatin zymography analysis.

## Methods

### Trabecular meshwork, cell culture, and identification

The trabecular meshwork tissues used in this study were obtained from three POAG patients (2 males and 1 female) aged 23 to 45 years who underwent trabecular surgery. The trabecular tissues were collected without the use of mitomycin C. Ethical approval was obtained from the Ethics Committee of Fujian Medical University, Quanzhou, China, and informed consent was obtained from each participant.

The details on cell culture method have been reported elsewhere [7]. In brief, the collected trabecular tissue was washed twice with Hank's solution before putting in a 25 ml plastic flask. The flask was then placed in a 5% CO_2_ incubator at 37 °C. After 10 min, a 20% concentration of fetal bovine serum (Hyclone, Logan, UT) was added and the flask was put back to the incubator. When local integration of the cells occurred, they were washed twice again with Hank's solution. Finally, a 0.25% concentration of trypsin (Hyclone Company) was added to the flask for cell subculture (0.1 ml/cm).

### Immunohistochemical study

The third-generation trabecular cells were grown to confluence on 9×9-mm cover slips in 24-well plates. And the cells were processed for indirect immunofluorescence analysis using a rabbit anti-human fibronectin (FN) monoclonal antibody, a mouse anti-human laminin (LM) monoclonal antibody, and a mouse anti-human neuron-specific enolase (NSE) monoclonal antibody (New Step Company, Fuzhou, China). Immunofluorescence staining of FN, LM, and NSE were visualized with a Nikon Eclipse TE2000-U inverted fluorescent microscope (Nikon, Tokyo, Japan).

### Transmission electron microscopy

The trabecular meshwork cultures were centrifuged and the supernatants were discarded, and the cells were then fixed with a mixture of 2.5% glutaraldehyde and 2% paraformaldehyde for observation by transmission electron microscopy (TEM; Hitachi H-7650, Tokyo, Japan).

### Hyaluronic acid (HA) treatment

The third generation trabecular meshwork cells (6×10^4^ cells/ml) were placed in a well plate, which was divided into 4 sections. Each section was treated with one of four HA concentrations (i.e., 0, 1, 3, or 6 mg/ml; Sigma Corporation, St Louis, MO) until the cells became confluent, and then further cultured without serum for 24 h before RT–PCR. The supernatant from the cell culture was also collected after 10 min centrifugation (1,000× g) for gelatin zymography analysis.

### Reverse transcription-polymerase chain reaction (RT–PCR)

The HA-treated trabecular meshwork cells were washed twice with Hank's solution, and placed at room temperature for 5 min after adding 1 ml of Trizol reagent (Invitrogen Corporation, Carlsbad, CA) to each well. Then, 200 μl of chloroform was added; the plates were shaken violently for 15 s and then left at room temperature for 3 min before centrifugation (12,000× g) at 4 °C for 15 min. The supernatant was transferred to an Eppendorf centrifuge tube (EP tube) colorless tube with 500 μl isopropanol, which was gently and evenly shaken, kept at room temperature for 10 min and then centrifuged at 12,000× g at 4 °C for 8 min to precipitate the total RNA. The supernatant was discarded and 1 ml of 75% alcohol (treated with DEPC water) was added to the tube. After another centrifugation at 7,500× g at 4 °C for 5 min, the supernatant was discarded. The pellets were left to dry for 3 to 5 min before adding 10 μl disinfected, DEPC treated, double-distilled water to provide the RNA samples for the study.

A UV spectrophotometer with a wavelength of 260/280 nm was used to determine RNA quantity and purity in a mixture sample of 1 μl RNA and 49 μl double-distilled water. If the absorbance ratio was 1.8 or more, 2 μg of the RNA was mixed with 1 μl OligoDT, with DEPC-treated water added to a final volume of 14.2 μl for the reverse transcription (RT). After centrifugation, the mixture was then incubated at 65 °C in a water bath for 5 min and then immediately place on ice before adding 10× AffinityScript™ RT Buffer (2 μl; Stratagene, Waldbronn, Germany), 100 Mm dithiothreitol (DTT; 2 μl), 100 mM dNTP mix (0.8 μl) and AffinityScript^TM^ Multiple Temperature Reverse Transcriptase (1 μl). After mixing gently, it was again incubated at 55 °C for one h and then bathed in 77 °C water for 10 min for the reaction termination. The synthesized cDNA was used for further quantization of mRNA.

The polymerase chain reaction (PCR) was performed according to the PCR kit instructions (Stratagene Corporation, Waldbronn, Germany): 2.0 μl cDNA was mixed with 10× PCR buffer (2.5 μl), 10 mmol/l dNTP mix (0.5 μl), upstream primer (Table 1) of 10 μmol/l (0.5 μl), downstream primer (Table 1) of 10 μmol/l (0.5 μl) and Taq enzyme of 5 unit/μl (0.3 μl) and then double-distilled water was added to a final volume of 25 μl. The amplification was conducted as follows: pre-denaturation for 4 min at 94 °C → denaturation for 30 s at 94 °C → annealing for 1 min at 55 °C for *MMP-2* and glyceraldehyde-3-phosphate dehydrogenase (*GAPDH*), 56 °C for *MMP-9* or 62 °C for β-actin (*ACTB*) → extension for 1 min at 72 °C → repeated 35 times → and a final extension for 10 min at 72 °C. A mixture of 8 μl of product from PCR and 3 μl PCR marker (Stratagene Corporation) was then subjected to gel electrophoresis for 40 min under 100V in 1.5% agarose gel containing ethidium bromide. The electrophoretic bands were quantified using a gel electrophoresis image analysis camera.

**Table 1:** Primer pairs used for Reverse transcription-polymerase chain reaction.

**Gene**	**Sense primer**	**Antisense primer**	**Probe (bp)**
*MMP2*	5’-GCGACAAGAAGTATGGCTTC-3’	5’-TGCCAAGGTCAATGTCAGGA-3’	390
*MMP9*	5’-AGTTCCCGGAGTGAGTTGAA-3’	5’-CTCCACTCCTCCCTTTCCTC-3’	196

### Gelatin zymography analysis

According to the zymography method kit manual (Genius USA Genetic Medicine Technology Company, Shanghai, China), substrate from gel electrophoresis (zymography) on gels containing gelatin was used. The liquid level was adjusted according to the number of cells. Standards of MMP-2 or MMP-9 (Sigma Corporation) were added to the substrate as a reference. Electrophoresis was continued until the dye reached the bottom of the gel. The gel was removed, treated with refolding solution, digestion solution, dye solution, and stained gradually, and then treated with bleaching solution until a clear section of white band appeared in dark blue background. Finally, the band area and gray scale were measured using the gel image analysis system. The content of the enzyme was calculated by multiplying the area of the band with the difference in gray scale between the band and the background.

### Statistical analysis

The means for the expressions of MMP-2 and MMP-9 by the two methods were reported at each HA concentration level. Linear regression with cluster and robust options was used to assess the effects of different HA concentrations on the expressions of MMP-2 and MMP-9 after adjusting for data correlation within patients. A p-value of less than 0.05 was considered as statistical significant. The analyses were performed using Stata 11 (Stata Corporation, College Station, TX).

## Results

**Figure 1:**
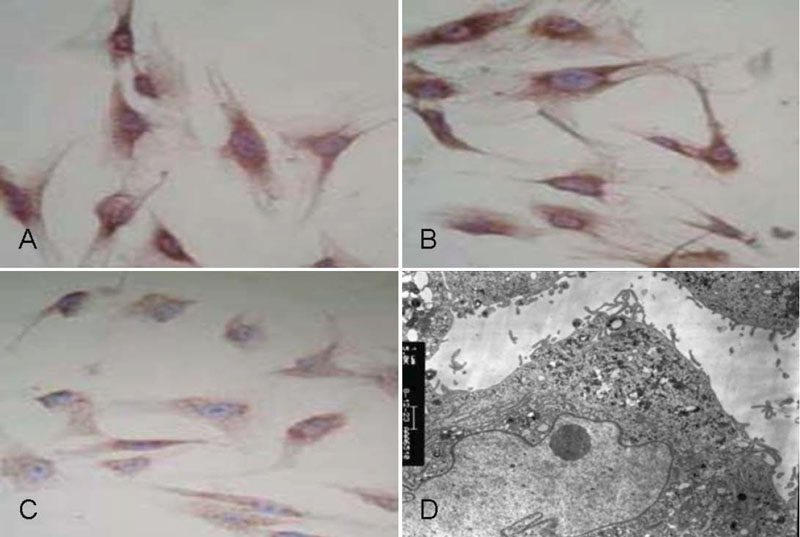
FN staining, LM staining, NSE staining, and TEM image of trabecular meshwork cells. **A**: Fibronectin (FN) staining (400×). **B**: Laminin (LM) staining (400×). **C**: Neuron-specific enolase (NSE) staining (5,000×). **D**: Transmission electron microscopy (TEM) image.

The trabecular cells were successfully cultured from trabecular tissues of the POAG patients using the tissue block method. Immunohistochemical staining for Fibronectin (FN; Figure 1A), laminin (LM) staining (Figure 1B), or neuron-specific enolase (NSE) staining (Figure 1C) were all positive. Transmission electron microscopy indicated that the cells were oval or round shape with plenty of surface microvilli. The major conjunctions of the cells are gap and tight connections. The cytoplasmic organelles were abundant as well as lysosomes, endoplasmic reticulum, mitochondria, phagocytic vesicles, and ribosomes (Figure 1D).

**Figure 2:**
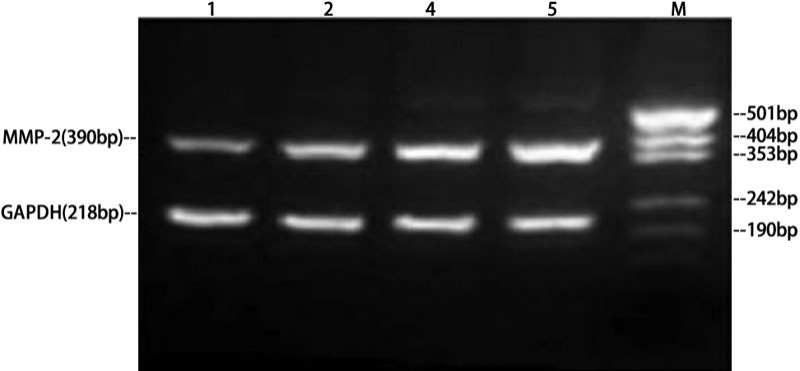
Gel electrophoresis of *MMP-2* mRNA and *GAPDH* mRNA PCR in cultured trabecular meshwork cells. MMP=Matrix metalloproteinases.

**Figure 3:**
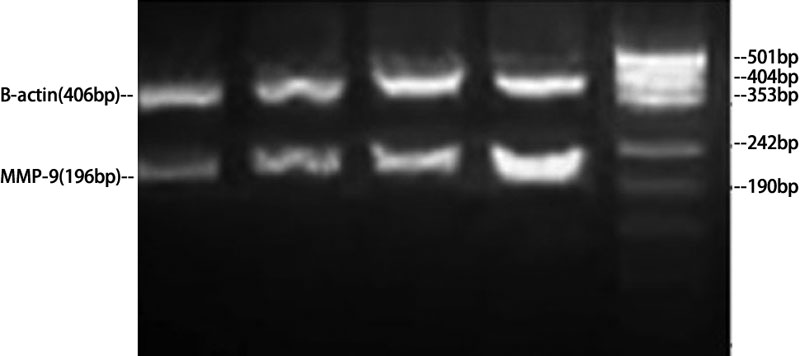
Gel electrophoresis of *MMP-9* mRNA and *ACTB* mRNA PCR in cultured trabecular meshwork cells. MMP=Matrix metalloproteinases.

**Figure 4:**
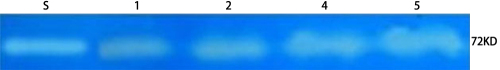
Spectral analysis of MMP-2 protein in trabecular meshwork supernatant. MMP=Matrix metalloproteinases.

**Figure 5:**
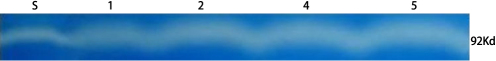
Spectral analysis of MMP-9 protein in trabecular meshwork supernatant. MMP=Matrix metalloproteinases.

**Figure 6:**
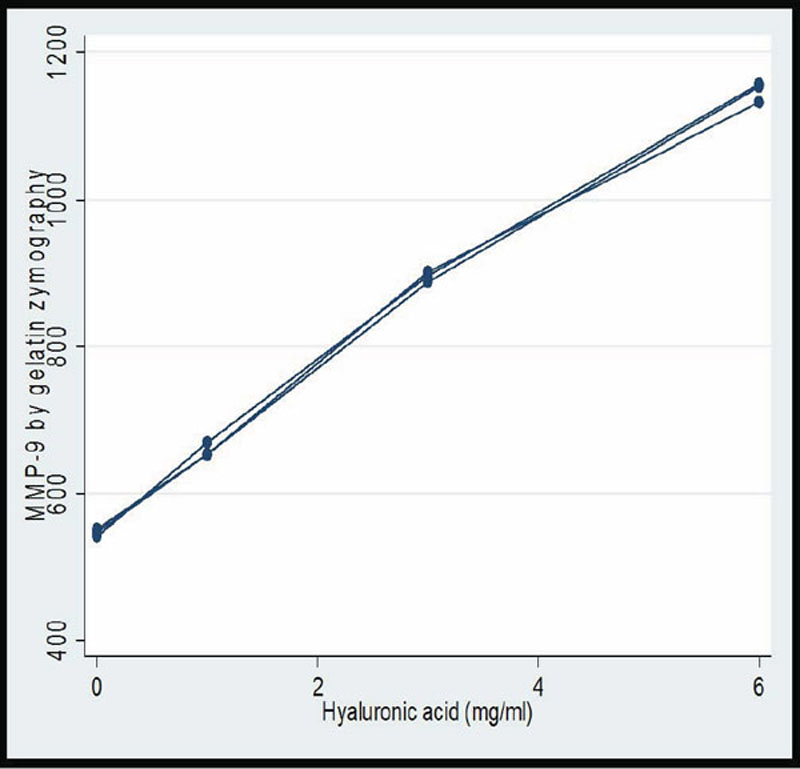
Gelatin zymography analysis showing a dose–response relationship of HA on the protein expression of MMP-9. MMP=Matrix metalloproteinases; HA=hyaluronic acid.

Table 2 presents the mRNA expression of *MMP-2* and *MMP-9* by RT–PCR (Figure 2 and Figure 3), and the protein expression of them by gelatin zymography analysis (Figure 4 and Figure 5) for the three POAG patients. All expressions increased with increasing HA concentration, indicating dose–response relationships between HA and the expressions of MMP-2 and MMP-9 in this range of HA concentration levels. These relationships appear to be linear. An approximately linear increase of the expression of MMP-9 by gelatin zymography analysis was observed in the HA concentration levels of 0 to 6 mg/ml (Figure 6). Therefore, HA was treated as a continuous variable in the regression analyses. According to the regression analyses, all the relationships are statistically significant at a level of p<0.001 (Table 3). Table 3 also presents the effect that sizes of HA had on the expression of MMP-2 and MMP-9 by RT–PCR and gelatin zymography analysis. There was a regression coefficient (effect size) of 100 for HA on the expression of MMP-9 by gelatin zymography analysis. This suggests that MMP-9 expression increased by 100 as per unit (mg/ml) increases in HA concentration based on the gel electrophoresis image analysis camera.

**Table 2:** Expression of MMP-2 and MMP-9 among the three POAG patients.

	MMP-2 (mg/ml) by RT–PCR*				**MMP-2 (mg/ml) by gelatin zymography**			
	**Hyaluronic acid**				**Hyaluronic acid**			
**POAG patients**	**0 mg/ml**	**1 mg/ml**	**3 mg/ml**	**6 mg/ml**	**0 mg/ml**	**1 mg/ml**	**3 mg/ml**	**6 mg/ml**
Patient 1	0.60	1.13	1.45	1.90	180	270	356	452
Patient 2	0.66	1.13	1.42	1.87	179	265	351	442
Patient 3	0.62	1.08	1.37	1.85	170	259	354	449
Mean	0.63	1.11	1.41	1.87	176	264	353	448
** **	MMP-9 (mg/ml) by RT–PCR*				**MMP-9 (mg/ml) by gelatin zymography**			
** **	**Hyaluronic acid**				**Hyaluronic acid**			
**POAG patients **	**0 mg/ml**	**1 mg/ml**	**3 mg/ml**	**6 mg/ml**	**0 mg/ml**	**1 mg/ml**	**3 mg/ml**	**6 mg/ml**
Patient 1	0.71	1.06	1.28	2.08	547	653	901	1132
Patient 2	0.62	1.08	1.38	2.05	552	653	887	1153
Patient 3	0.69	1.15	1.40	2.12	541	669	896	1156
Mean	0.67	1.10	1.35	2.08	547	659	895	1147

* Reverse transcription-polymerase chain reaction. MMP=Matrix metalloproteinases; POAG=Primary open-angle glaucoma.

**Table 3:** Effects of HA on the expressions of MMP-2 and MMP-9.

**Regression models**	**Constant**	Coeff* (95% CI) for HA	**p**
Model 1 (MMP-2 by RT–PCR*)	0.777	0.192 (0.175–0.209)	<0.001
Model 2 (MMP-2 by gelatin zymography)	202.7	43.1 (39.3–47.0)	<0.001
Model 3 (MMP-2 by RT–PCR*)	0.749	0.221 (0.212–0.230)	<0.001
Model 4 (MMP-2 by gelatin zymography)	560.9	100.3 (95.7–104.9)	<0.001

Hyaluronic acid (HA) is considered as a continuous variable in linear regression model with cluster (patient) and robust options. MMP=Matrix metalloproteinases.

## Discussion

This study shows that trabecular meshwork cells in vitro can express MMP-2 and MMP-9, key regulators of ECM. The mRNA and protein expression of MMPs were increased by increasing HA concentrations. Isnard et al. [8] also reported increased expression of MMP-2 and MMP-9 after HA treatment in the corneal cells in vitro. To our knowledge, the effect of HA on trabecular meshwork cells has not been reported previously. High levels of matrix metalloproteinases have been demonstrated to clear the deposition of ECM in the trabecular meshwork and thus facilitate the outflow of aqueous humor [9-11]. Therefore, our results are in line with our hypothesis that a reduction in activities of MMPs may result from a lack of HA in aqueous humor, which might increase the resistance of aqueous outflow and intraocular pressure. Further studies are needed to determine whether HA level in TM/aqueous might be used as a marker to evaluate the prognosis of IOP lowering treatment among glaucoma patients.

It remains unclear how the expression of MMPs is regulated by HA in POAG eyes. In our previous experiment, we have shown that cultured trabecular meshwork cells from normal human eyes expressed CD44 molecules [12]. A large number of CD44 molecules are also expressed in lung cancer cells [13]. The secretion of MMP-2 and MMP-9 in the cancer cells can be promoted by HA treatment, and this is believed to be related to the HA-CD44-Ras-MEK1-MAPK signaling pathway because the effect of HA could be inhibited by anti-CD44 antibody, Manumycin A and mitogen-activated protein kinase (MAPK) or mitogen-activated protein kinase kinase-1 (MEK-1) inhibitor (PD98059) [13]. Miletti et al. [14] also reported that CD44s was involved in the regulated expression of MMPs and contributed to a higher metastatic potential and aggressive phenotype. Further studies are needed to examine whether the HA-CD44-Ras-MEK1-MAPK signaling pathway is responsible for the effects of HA found in this study. Future study is also needed to compare the effect of HA level on MMP expression between POAG patients and normal controls.

A large number of studies have shown that MMPs are the key players in the regulation of trabecular outflow, which directly affects intraocular pressure. Depletion of ECM degrading enzyme and increasing activities of the trabecular meshwork cells synthesis and secretion may lead to an accumulation of the ECM in the outflow facilities and an increase of the aqueous outflow resistance [5,15,16]. In an experiment using a human eye perfusion model, Bradley et al. [15], found that increasing metalloproteinase activity by adding MMP-2, MMP-9, and MMP-3 to the culture medium of anterior segment tissue increased the outflow rate by 160%, while in opposite, an inhibition of endogenous trabecular metalloproteinase activity using TIMPs might reduce the outflow rate by 40%. More interestingly, such increase or decrease of outflow is reversible. They believed that at least in this human culture model, endogenous ECM turnover by these enzymes was required for the maintenance of trabecular outflow resistance. In another study by Bradley et al. [16], increased expression and secretion of both interleukin-1b (IL-1b) and tumor necrosis factor-α (TNF-α) were observed in the first 8 h after laser trabeculoplasty. These cytokines mediate an increase of trabecular stromelysin expression, initiate the remodeling of the juxtacanalicular extracellular matrix (a likely site for the aqueous outflow resistance) and finally lead to a restoration of normal outflow facility. Kelley et al. [17] also found that IL-1 and TNF-α had synergistic effect on the expression of MMP-3 and MMP-9 in human trabecular meshwork cells. Schlotzer-Schrehardt et al. [5] suggested that reduced MMP activity in aqueous humor as well as a change in the local MMP-TIMP balance may promote the abnormal matrix accumulation characteristic of pseudoexfoliation syndrome and be causally involved in the pathogenesis of both pseudoexfoliation glaucoma and POAG.

In conclusion, the expressions of MMP-2 and MMP-9 in the trabecular meshwork cells are at least in part regulated by HA in POAG eyes. Therefore, a depletion of HA can reduce the activities of MMP-2 and MMP-9 in aqueous humor that may cause an accumulation of extra ECM in the outflow facilities and thus increase the aqueous outflow resistance and eventually result in an increase of intraocular pressure. This pathway may be involved in the pathogenesis of POAG and deserves further attention in the future glaucoma research.
